# Preparation and Characteristics of Na_2_HPO_4_·12H_2_O-K_2_HPO_4_·3H_2_O/SiO_2_ Composite Phase Change Materials for Thermal Energy Storage

**DOI:** 10.3390/ma15217600

**Published:** 2022-10-29

**Authors:** Rongda Ye, Jun Wang, Yanna Li, Wanchun Sun, Qizhang Huang, Sheng Gong, Xugang Shu

**Affiliations:** 1 School of Chemistry and Chemical Engineering, Zhongkai University of Agriculture and Engineering, Guangzhou 510225, China; 2 Shunde Polytechnic, Foshan 528300, China

**Keywords:** thermal energy storage, building energy conservation, composite phase change materials, fumed silica, eutectic hydrated salts

## Abstract

In this paper, a series of eutectic hydrated salts was obtained by mixing Na_2_HPO_4_·12H_2_O (DHPD) with K_2_HPO_4_·3H_2_O (DHPT) in different proportions. With the increase in the content of DHPT, the phase transition temperature and melting enthalpy of eutectic hydrated salts decreased gradually. Moreover, the addition of appropriate deionized water improved the thermal properties of eutectic hydrated salts. Colloidal silicon dioxide (SiO_2_) was selected as the support carrier to adsorb eutectic hydrated salts, and the maximum content of eutectic hydrated salts in composite PCMs was 70%. When the content of the nucleating agent (Na_2_SiO_3_·9H_2_O) was 5%, the supercooling degree of composite PCMs was reduced to the minimum of 1.2 °C. The SEM and FT-IR test results showed that SiO_2_ and eutectic hydrated salts were successfully combined, and no new substances were formed. When the content of DHPT was 3%, the phase transition temperature and melting enthalpy of composite PCMs were 26.5 °C and 145.3 J/g, respectively. The results of thermogravimetric analysis and heating–cooling cycling test proved that composite PCMs had good thermal reliability and stability. The application performance of composite PCMs in prefabricated temporary houses was investigated numerically. The results indicated that PCM panels greatly increased the Grade I thermal comfort hours and reduced energy consumption. Overall, the composite PCM has great development potential building energy conservation.

## 1. Introduction

Global greenhouse gas emissions will bring serious problems such as environmental pollution [1]. According to research reports, greenhouse gas emissions are mainly caused by the large consumption of energy [2,3]. Therefore, it is necessary to vigorously develop advanced energy storage technology. The thermal energy storage system is generally divided into thermochemical energy storage, sensible heat storage, and latent heat storage. The energy storage density of the thermochemical energy storage system is the largest, but it has the disadvantages of high cost, uncontrollable process, and strict requirements for equipment [4]. The sensible heat storage system is simple, but it has the defects of large device volume and low energy storage density [5]. The latent heat storage system releases and stores heat through the phase transition process of the phase change materials (PCMs) [6]. When the temperature exceeds the phase transition temperature, the surrounding heat will be absorbed and stored by PCMs. When the temperature is lower than the phase transition temperature, the heat stored by PCMs will be released. Due to its advantages of easy control and high energy storage density, it has been widely concerned by researchers [7,8].

PCMs are generally classified into organic, inorganic, and eutectic PCMs. Paraffins and fatty acids are the most common organic PCMs, which have been widely studied because of their good chemical and thermal stability [9,10]. Wu et al. [11] used the three-dimensional structure of cellulose-based carbon aerogel to adsorb stearic acid. The results indicated that the composite PCM had high light–thermal conversion efficiency. Li et al. [12] investigated the adsorption effect of mesoporous silica with different pore sizes on paraffin. It was found that the silica with the smallest pore diameter had the largest adsorption capacity for paraffin. However, organic PCMs are generally flammable and expensive, which hinders their practical application [13]. Inorganic PCMs mainly include metals, molten salts, and hydrated salts. Due to the low phase transition temperature and wide application range of inorganic hydrated salts, it has received more attention at present [14,15]. Inorganic hydrated salts, such as calcium chloride hexahydrate, sodium carbonate decahydrate, sodium acetate trihydrate, disodium hydrogen phosphate dodecahydrate, etc., come from a wide range of sources. The properties of low cost, high latent heat, and non-flammable make them popular, but they have defects such as supercooling and phase separation that need to be improved [16]. Jin et al. [17] found that a mixed nucleating agent composed of graphene oxide and strontium chloride hexahydrate could decrease the supercooling degree of calcium chloride hexahydrate from 25.4 °C to 0.3 °C. After 200 cooling and heating cycles, the supercooling degree was still below 1 °C. Deng et al. [18] used expanded vermiculite to compound with disodium hydrogen phosphate dodecahydrate. By adding 5.3 wt% aluminum as a nucleating agent, its supercooling degree was reduced to 1.4 °C. The eutectic PCM is obtained by combining two or more PCMs. For a single PCM, its phase change properties may sometimes not meet the requirements. The desired phase transition temperature can be obtained by mixing different PCMs in a certain proportion to obtain a eutectic PCM [19,20]. Wang et al. [21] obtained a PCM with a phase transition temperature of 20~25 °C, which was achieved by adding 0.5% hydroxyethyl cellulose, 5% SrCl_2_·6H_2_O, and 35% MgCl_2_·6H_2_O into a solution of CaCl_2_·6H_2_O. The change in the phase transition temperature of CaCl_2_·6H_2_O was due to the formation of eutectic salts after adding MgCl_2_·6H_2_O. Li et al. [22] added urea to a mixed solution of CH_3_COONa·3H_2_O and potassium chloride. According to the characteristic that urea could form a eutectic salt with CH_3_COONa·3H_2_O, a PCM with a phase transition temperature suitable for the heat pump system was obtained. Liu et al. [23,24] mixed Na_2_HPO_4_·12H_2_O with Na_2_SO_4_·10H_2_O and Na_2_CO_3_·10H_2_O, respectively. When the content of Na_2_SO_4_·10H_2_O was 25%, the eutectic hydrated salt with the lowest melting temperature was obtained, which was 31.2 °C. For Na_2_CO_3_·10H_2_O, when its content was 40%, there was only one peak on the DSC melting curve. That was to say, Na_2_HPO_4_·12H_2_O and Na_2_CO_3_·10H_2_O formed a eutectic hydrated salt at this time, and its phase transition temperature and melting enthalpy were 27.3 °C and 220.2 J/g, respectively. The phase transition temperature of Na_2_HPO_4_·12H_2_O is about 35 °C, which has the advantages of large enthalpy and no phase separation [25]. It is expected to combine with other PCMs to form temperature-tunable eutectic PCMs. The emergence of eutectic PCMs broadened the application range of PCMs.

For eutectic hydrated salts, they have the problem of liquid leakage during practical application. The use of porous materials to adsorb PCMs into the pore structure has been proven to be a simple and effective solution to the problem of liquid leakage [26,27,28]. Wu et al. [29] selected expanded graphite to adsorb eutectic hydrated salts. It was found that expanded graphite not only prevented the leakage of hydrated salts, but also reduced the supercooling degree of hydrated salts. Fu et al. [28] used fumed silica as the support carrier and temperature regulator to prepare composite PCMs with suitable phase transition temperature and stable performance. Liu et al. [30] obtained a new type of composite PCMs by adsorbing hydrated eutectic salts into the pore structure of fly ash through a simple impregnation method. Among many porous materials, silicon dioxide has both lipophilic and hydrophilic types, which is suitable for adsorbing various PCMs [31]. In addition, silicon dioxide has a high specific surface area and rich pore structure. Compared with porous carriers such as expanded vermiculite and expanded perlite, it can absorb more PCMs, so its composite PCMs have better thermal properties [32,33]. At present, there are few reports on the research of eutectic hydrated salts/ silicon dioxide composite PCMs.

In this paper, a series of eutectic hydrated salts were obtained by mixing dipotassium hydrogen phosphate trihydrate (DHPT) and disodium hydrogen phosphate dodecahydrate (DHPD) with different contents. Colloidal silicon dioxide (SiO_2_) was selected to adsorb eutectic hydrated salts. Sodium metasilicate nonahydrate was used as the nucleating agent to reduce the supercooling degree of composite PCMs. The properties of composite PCMs were tested and characterized, including the supercooling degree, phase change characteristics, micromorphology, chemical composition, thermal stability, and thermal reliability. The application of composite PCMs in prefabricated temporary houses was investigated through numerical simulation. In this work, composite PCMs based on eutectic hydrated salts with good performance were obtained, and their application potential in the construction field was demonstrated.

## 2. Experimental Section

### 2.1. Materials

Dipotassium hydrogen phosphate trihydrate (DHPT, purity > 99%) and sodium metasilicate nonahydrate (Na_2_SiO_3_·9H_2_O, purity > 99%) were purchased from Sinopharm Chemical Reagent Co., Ltd. (Shanghai, China). Disodium hydrogen phosphate dodecahydrate (DHPD, purity > 99%) was purchased from Shanghai Aladdin Biochemical Technology Co., Ltd. (Shanghai, China). Colloidal silicon dioxide (SiO_2_) was obtained from Shandong Yousuo Chemical Technology Co., Ltd. (Linyi, China)

### 2.2. Preparation Process of Eutectic Hydrated Salts

Firstly, a certain amount of DHPD and DHPT were weighed and put into a sealable glass bottle. Then, a certain proportion of deionized water was dropped into the glass bottle. In addition, Na_2_SiO_3_·9H_2_O was used as the nucleating agent to reduce the supercooling degree of PCMs. Finally, the eutectic hydrated salt solution was obtained by stirring them uniformly at 50 °C. The eutectic hydrated salt with different phase transition temperatures could be prepared by adding DHPT with different mass fractions.

### 2.3. Preparation Process of Composite PCMs

To improve the liquid leakage phenomenon of solid–liquid PCMs, colloidal silicon dioxide was employed as the porous material to absorb the eutectic hydrated salt. First, SiO_2_ with different mass fractions was added to the eutectic hydrated salt solution prepared above. After stirring for 3 min, it was placed in an environment of 50 °C to melt the PCM, and then it was taken out and stirred again. This process was repeated several times to ensure that the PCM was fully adsorbed by SiO_2_. Finally, the composite PCM was obtained by cooling it at room temperature, and then it was put into the refrigerator for future use.

### 2.4. Characterization

**Figure 1:**
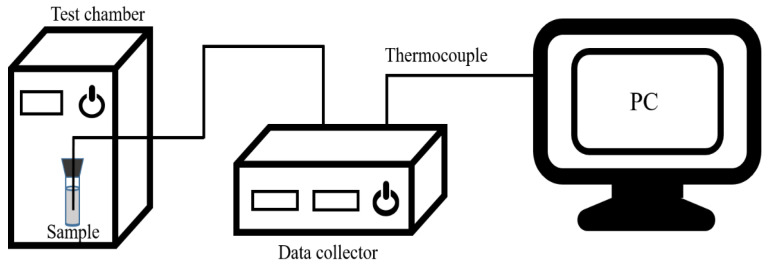
Schematic diagram of the experimental device for supercooling degree test.

The supercooling degree of the material was tested by the T-history method, and the simple diagram of the testing device is displayed in Figure 1. The samples were poured into a sealed glass tube and compacted, and then placed in a test chamber (BPHJS-060B, Shanghai Yiheng Technology Instrument Co., Ltd., Shanghai, China) that could adjust the temperature and humidity. A K-type thermocouple was inserted and fixed at the center of the sample. Agilent 34970A data acquisition instrument was used to record the change in sample temperature with time.

The liquid leakage test was used to determine the appropriate content of eutectic hydrated salts in composite PCMs. The composite PCMs containing eutectic hydrated salts of different mass fractions were pressed into blocks and placed on clean filter papers. They were heated in an oven at 50 °C for 30 min and then taken out to carefully observe whether there were water stains.

The scanning electron microscope (SEM, Zeiss Gemini 500, Jena, Germany) was selected to observe the micromorphology of the composite PCM and SiO_2_. Fourier transform infrared spectroscopy (FT-IR, Nicolet iN10, Waltham, MA, USA) was employed to test the chemical composition of the sample. The thermal characteristics of the sample, such as phase transition temperature and melting enthalpy, were characterized by the differential scanning calorimeter (DSC, NETZSCH 214 Polyma, Selb, Germany). The thermogravimetric analyzer (TGA, Mettler Toledo TGA2, Columbus, OH, USA) was selected to test the thermal decomposition temperature and thermal stability of the materials. For DSC and TG tests, the sample mass was about 10 mg. The test was conducted at a heating rate of 5 °C/min under a nitrogen atmosphere.

A heating–cooling cycling test was employed to evaluate the thermal reliability of the composite PCM. A small amount of the composite PCM was poured into a sealable bottle, and then it was put into a test chamber with adjustable temperature and humidity (BPHJS-060B, Shanghai Yiheng Technology Instrument Co., Ltd., Shanghai, China). For the temperature procedure of the test chamber, it took 10 min to lower the temperature to 0 °C, and then the sample was kept constant at 0 °C for 20 min. Then, it took 10 min to raise the temperature to 50 °C, and then the sample was kept constant at 50 °C for 20 min. The whole process was repeated 200 times.

## 3. Results and Discussion

### 3.1. Supercooling Degree

**Figure 2:**
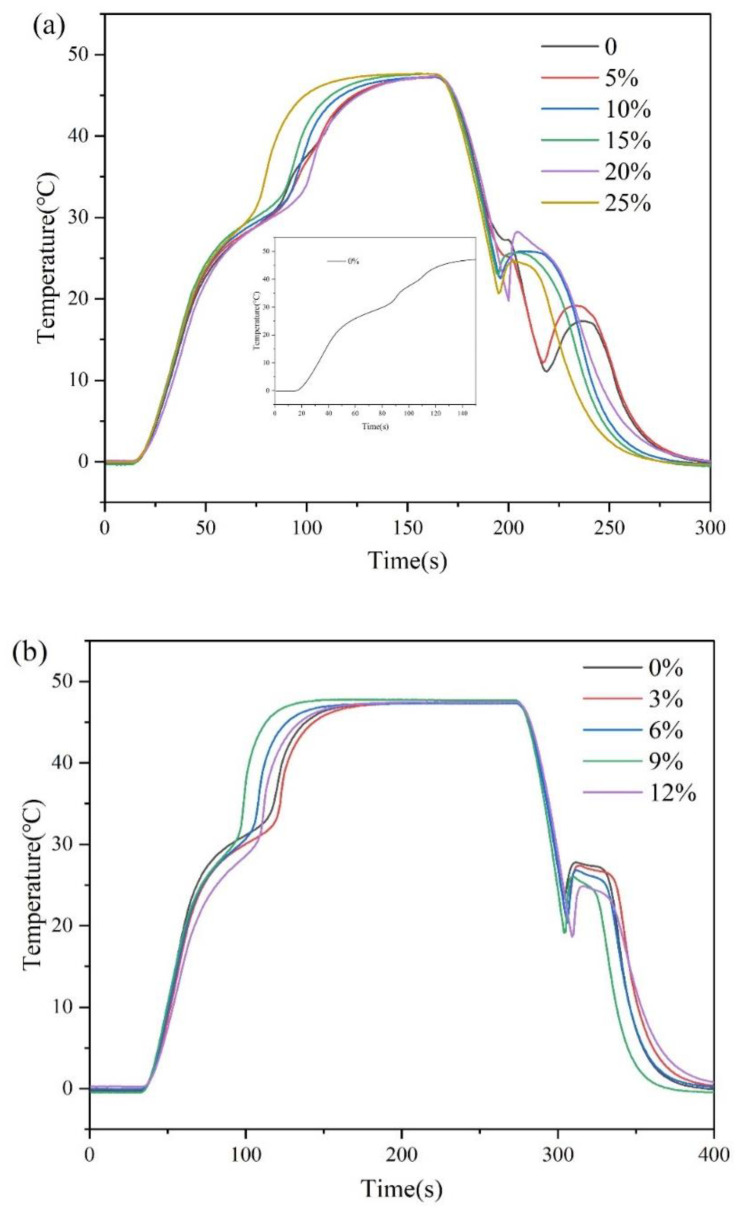

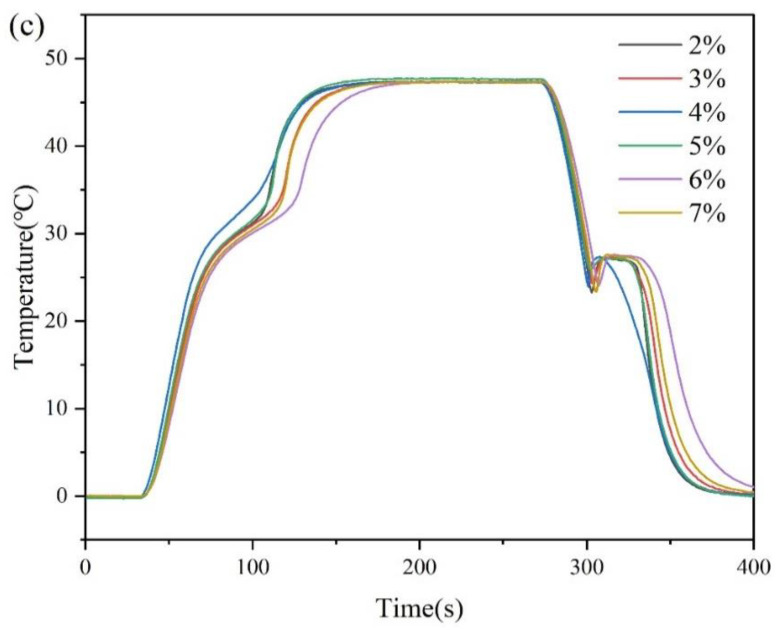
Cooling curves: (**a**) eutectic hydrated salts with different deionized water content; (**b**) COMPOSITE PCMs with different DHPT content; (**c**) composite PCMs with different Na_2_SiO_3_·9H_2_O content.

The cooling curves of the eutectic hydrated salt with different mass fractions of deionized water are displayed in Figure 2a, and the corresponding supercooling degree is shown in Table 1. It could be seen that the melting process of eutectic hydrated salts was divided into two stages when no deionized water was added. This may be related to the inherent defect that DHPD is easy to lose crystal water, which will lead to the decline of the thermal storage performance of eutectic hydrated salts. In this work, it was found that this phenomenon could be improved by adding deionized water to the material preparation process. When the mass fraction of deionized water was 15%, the supercooling degree of the sample was the minimum, which was 2.8 °C. Subsequently, the deionized water content was fixed to 15% of the mass of eutectic hydrated salts.

The cooling curve of the composite PCM is displayed in Figure 2b. When the content of DHPT was 0, 3, 6, 9, and 12% of the mass of eutectic hydrated salts, the supercooling degree was 4.5, 5.4, 6.4, 6.9, and 6.2 °C, respectively. The results showed that the combination of eutectic hydrated salts and SiO_2_ could not play a positive role in decreasing the supercooling degree. Moreover, with the gradual increase in the content of DHPT, the melting platform of composite PCMs became smaller, which meant that its heat storage capacity decreased. It could also be found that the initial melting temperature of the composite PCM decreased with the increase in the content of DHPT. For building application, the mass fraction of DHPT was determined to be 3% by comprehensively considering the heat storage capacity and phase transition temperature.

The cooling curve of the composite PCM containing the nucleating agent is displayed in Figure 2c. The results showed that the supercooling degree of the composite PCM decreased when Na_2_SiO_3_·9H_2_O was added as the nucleating agent. When the content of Na_2_SiO_3_·9H_2_O was 2, 3, 4, 5, 6, and 7% of the mass of eutectic hydrated salts, the supercooling degree was 3.7, 4.0, 3.4, 1.2, 3.6, and 4.2 °C, respectively. Therefore, when the mass fraction of Na_2_SiO_3_·9H_2_O was 5%, it was most helpful in reducing the supercooling degree of composite PCMs.

### 3.2. Optimum Content of Eutectic Hydrated Salts

**Figure 3:**
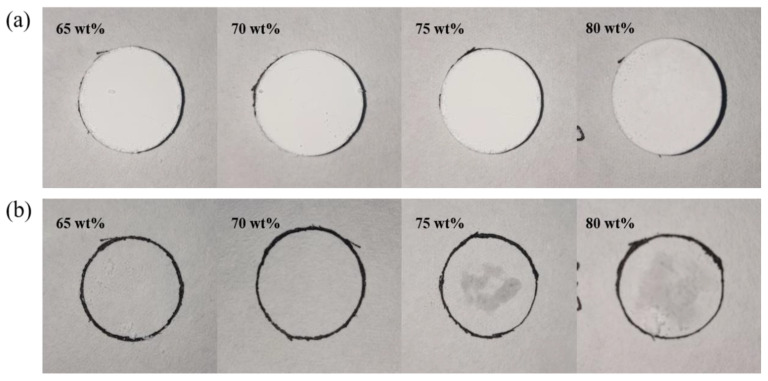
Liquid leakage test of composite PCMs: (**a**) before heating; (**b**) after heating.

The liquid leakage test results of the composite PCM are displayed in Figure 3. It could be seen from Figure 3a that composite PCMs could be pressed into blocks and shaped. After the composite PCMs were heated, the eutectic hydrated salts melted. It could be found from Figure 3b that when the content of eutectic hydrated salts was 65% or 70%, no trace of liquid leakage was observed on the filter paper. With the gradual increase in the content of eutectic hydrated salts, such as 75% or more, more and more water stains appeared. This phenomenon is mainly determined by the pore structure inside the supporting material. When the content of eutectic hydrated salts was too large, the pore volume of SiO_2_ could not adsorb all PCMs, which led to the leakage of eutectic hydrated salts during melting. Therefore, based on the experimental results, the optimal content of eutectic hydrated salts in the composite PCMs was about 70%.

### 3.3. Effects of DHPT on Phase Transition Characteristics

**Figure 4:**
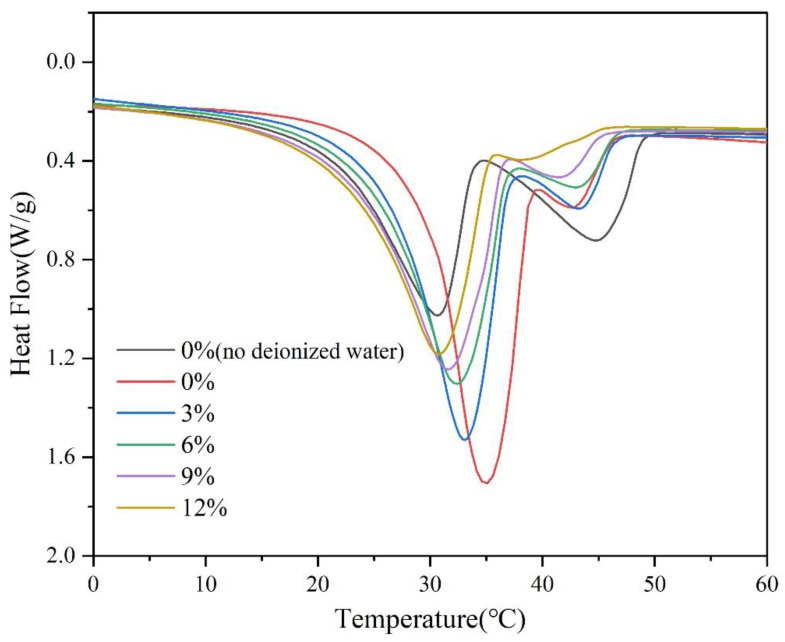
DSC curves of composite PCMs containing different contents of DHPT.

The change in phase transition temperature and melting enthalpy of composite PCMs with the content of DHPT is shown in Figure 4 and Table 2. When no additional deionized water was added during the preparation of composite PCMs, two broad peaks could be seen in the DSC curve, which might be related to the loss of crystal water from hydrated salts. At this time, the thermal properties of the composite PCM were poor, and its melting enthalpy was only 129.9 J/g. After adding the proper amount of deionized water in the preparation process, it could be observed that the second peak on the DSC curve was significantly reduced, which was conducive to improving the thermal properties of the composite PCM. When adding DHPT to DHPD, the phase transition temperature and melting enthalpy of composite PCMs decreased, which was consistent with the results of the cooling curves. As the mass fraction of DHPT increased from 0% to 12%, the melting enthalpy decreased from 150.3 J/g to 122.4 J/g, and the phase transition temperature dropped from 29.6 °C to 22.9 °C. When the content of DHPT was 3%, the melting enthalpy (145.3 J/g) of composite PCMs decreased slightly, and the phase transition temperature (26.5 °C) was suitable for building applications, so it was selected for subsequent research.

### 3.4. Morphology and Structure

**Figure 5:**
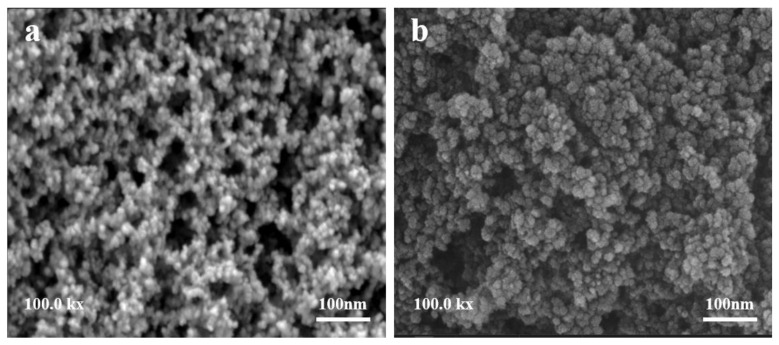
SEM images of SiO_2_ (**a**) and composite PCMs (**b**).

The micro morphology of the composite PCM and SiO_2_ is presented in Figure 5. It could be seen from Figure 5a that SiO_2_ molecules agglomerated into a three-dimensional cluster structure through intermolecular force. These abundant pore structures enabled it to well adsorb eutectic hydrated salts. It was observed from Figure 5b that the particles became larger. Through the capillary force and hydrophilic groups (such as hydroxyl) of SiO_2_, the eutectic hydrated salts were adsorbed in the pore structure and on the particle surface. Therefore, SiO_2_ could prevent the leakage of eutectic hydrated salts when phase transition occurred.

**Figure 6:**
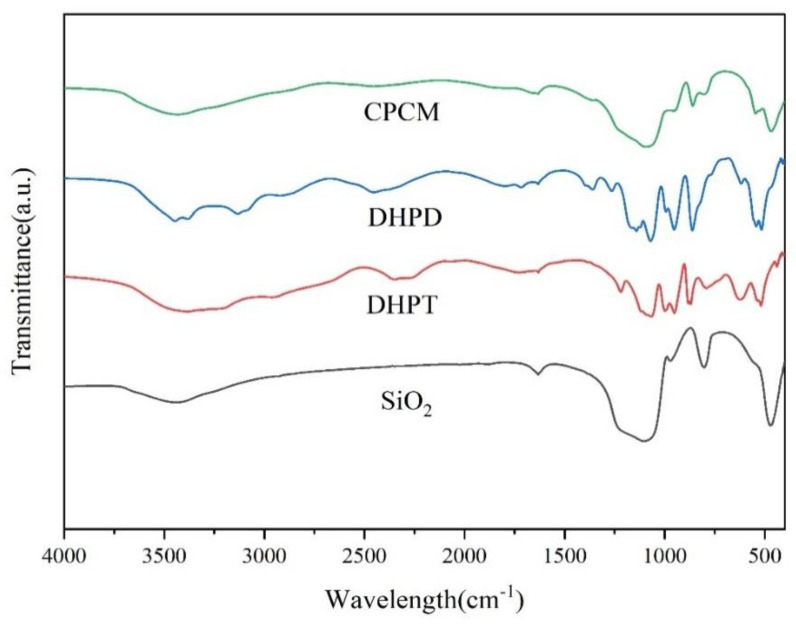
FT-IR spectra of SiO_2_, DHPT, DHPD, and composite PCMs.

The FT-IR spectrum of SiO_2_, DHPT, DHPD, and composite PCMs is presented in Figure 6. For the spectra of composite PCMs (CPCM), the characteristic absorption peaks at 1096 cm^−1^ and 4667 cm^−1^ correspond to the antisymmetric stretching vibration and bending vibration of Si-O-Si, respectively. The absorption peak at 1096 cm^−1^ was also the stretching vibration peak of HPO_4_^2−^. In addition, the stretching vibration peak of HPO_4_^2−^ also appeared at 859 cm^−1^ and 544 cm^−1^. Only the characteristic absorption peaks of HPO_4_^2−^ and SiO_2_ appeared in the composite PCMs, and no other peaks appeared. The results indicated no chemical reaction between eutectic hydrated salt and SiO_2_, but only physical adsorption.

### 3.5. Thermal Stability

**Figure 7:**
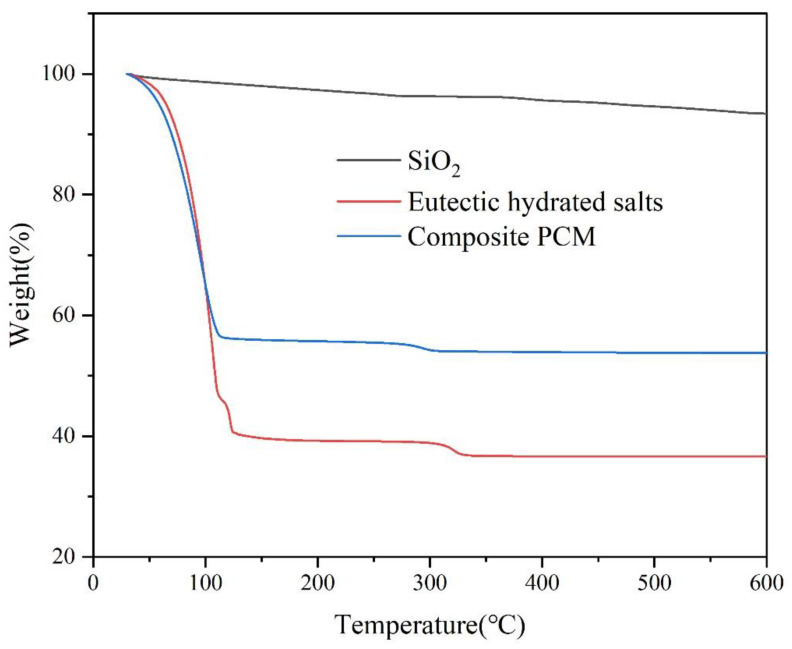
TGA curves of SiO_2_, eutectic hydrated salts, and composite PCMs.

The TGA curves of SiO_2_, eutectic hydrated salts, and composite PCMs are shown in Figure 7. Because SiO_2_ has hygroscopicity, it could be seen from the figure that it had weight loss during the process of heating to 600 °C. The weight loss of SiO_2_ was the water content in SiO_2_, which was 6.57%. For the eutectic hydrated salt, its weight loss came from the evaporation of crystal water. When the sample temperature was higher than 330 °C, the weight of eutectic hydrated salts basically did not decrease, and the total weight loss was 63.35%. For composite PCMs, the weight loss process was similar to that of the eutectic hydrated salt, and its total weight loss was 46.22%. According to the weight loss of SiO_2_, eutectic hydrated salts, and composite PCMs, it could be calculated that the content of eutectic hydrated salts in composite PCMs was about 69.8%, which was consistent with the theoretical value of 70%.

### 3.6. Thermal Reliability

**Figure 8:**
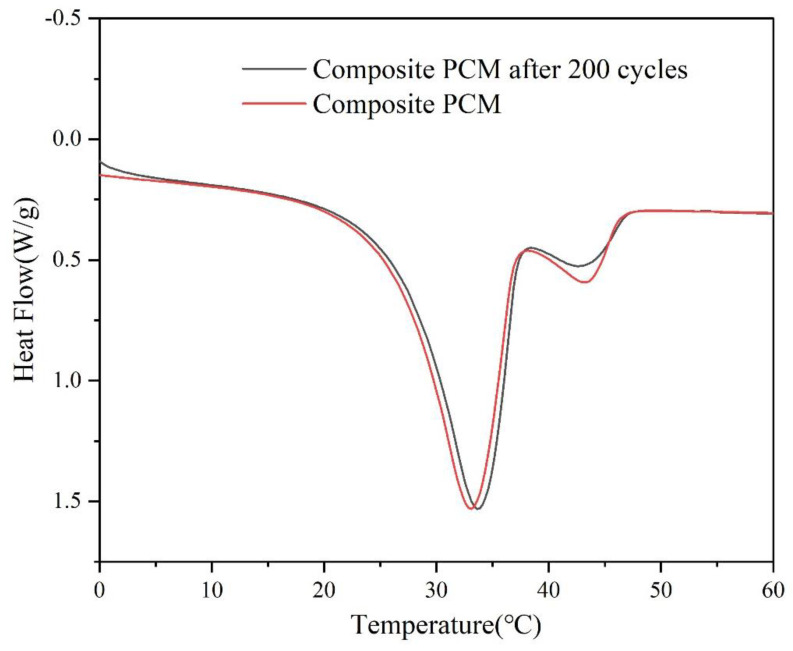
DSC curves of the composite PCM before and after 200 cycles.

The DSC curve and corresponding thermal characteristics of composite PCMs after 200 heating and cooling cycles are displayed in Figure 8 and Table 3. It could be seen that after 200 heating and cooling cycles, the thermal properties of composite PCMs had only changed a little. The phase transition temperature and the peak temperature of composite PCMs increased by about 0.6 °C. The melting enthalpy of the composite PCMs decreased from 145.3 J/g to 139.3 J/g. The results showed that the defects, such as phase separation, were improved, and it had good thermal stability. It can be imagined that the thermal properties of composite PCMs will not be seriously damaged during long-term use. Other similar studies were summarized in Table 4. After comprehensive comparison, the composite PCMs prepared in this study had high latent heat, so they have good potential in thermal energy storage applications.

## 4. Application Performance in a Prefabricated Temporary House

### 4.1. Physical Model

**Figure 9:**
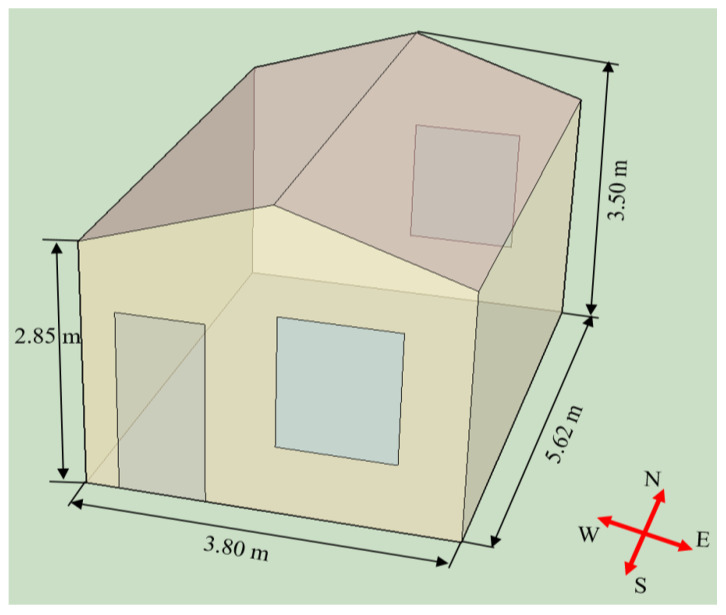
Schematic diagram of the prefabricated temporary house.

To study the building energy-saving potential of composite PCMs, the impact of composite PCMs on the thermal performance of prefabricated temporary houses was evaluated. The model diagram of the room is shown in Figure 9. The length, width, and height of the building model were 5.62, 3.80, and 2.85 m, respectively. There was a window with a width of 1.2 m and a height of 1.5 m on the north and south walls. There was also a door with a width of 0.9 m and a height of 2.1 m on the south wall. The roof of prefabricated temporary houses was a double-pitch roof. The geometry of the room model in this paper represented the typical traditional prefabricated temporary house in the current Chinese market. The structural details of prefabricated temporary houses and the thermophysical properties of materials involved in the building model are presented in Table 5 and Table 6, respectively. In this section, the prefabricated temporary house with and without PCM panels was named PCM rooms and reference rooms, respectively.

Energyplus v9.2 was selected as a tool for numerical simulation. The heat balance algorithm of conduction finite difference was adopted to simulate the PCM. The Chinese Standard Weather Data of Beijing, which is located in the cold region, was selected as the external environment. Because the thickness of the steel sheet was too thin, it needed to be ignored in the simulation. The infiltration ventilation rate and time step were set to 0.5 air change per hour (ACH) and 3 min, respectively. From the perspective of energy saving, the air conditioning temperature in the cooling season (15 June–15 September) and heating season (15 November–15 March) were set to 18 and 26 °C, respectively. The “HVAC Template: Zone: Ideal Loads Air System” object was selected to simulate the energy consumption of prefabricated temporary houses.

The predicted mean vote and predicted percentage of dissatisfied models (PMV-PPD) were employed to evaluate the residential thermal comfort of prefabricated temporary houses. The metabolic rate and air velocity were considered to be 1.0 met and 0.15 m/s, respectively. The clothing level was set at 0.5 clo from May to September, and 1.0 clo for other months. The number of people in prefabricated temporary houses was considered as 2, and the working efficiency value was set as 0. Based on the evaluation standard for the indoor thermal environment in civil buildings (GB/T 50785-2012), indoor thermal conditions are classified into three categories. As the PPD and absolute value of PMV become smaller, the thermal comfort becomes better, as shown in Table 7.

### 4.2. Analysis of Thermal Performance

**Figure 10:**
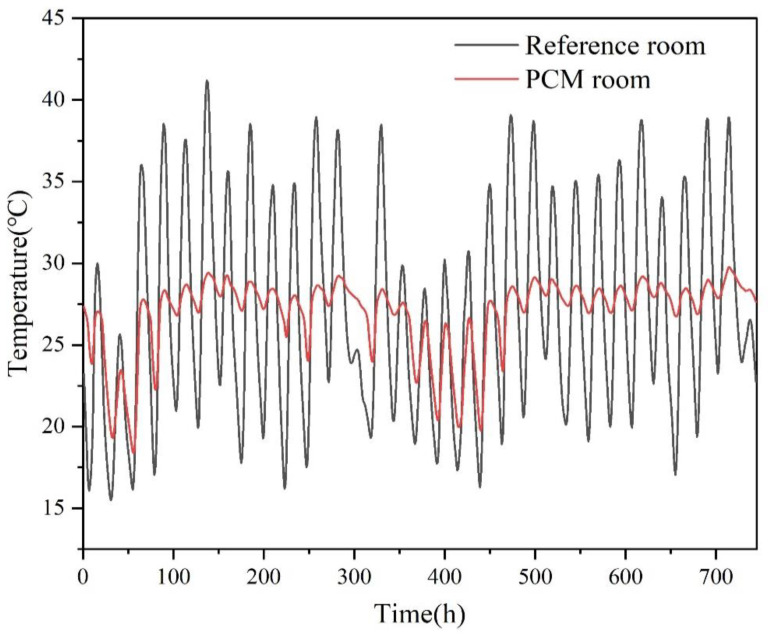
Indoor thermal comfort operative temperature of prefabricated temporary houses in May.

The thermal comfort hours and energy consumption of prefabricated temporary houses are shown in Table 8. For the reference room, the thermal comfort hours of Grade I, Grade II, and Grade III were 2187, 945, and 5498 h, respectively. When the 10 mm rock wool was replaced by the 10 mm PCM panel, the thermal comfort hours of Grade I and Grade II increased by 804 and 190 h, respectively. This meant that the PCM panel could play a positive role, which was better than the rock wool. The indoor temperature of prefabricated temporary houses in May is displayed in Figure 10. For the reference room, the room temperature was between 15 and 40 °C. Such a large temperature fluctuation made the thermal environment of prefabricated temporary houses unable to meet people’s requirements. For the PCM room, PCMs could absorb the heat transferred from the outside in the daytime, which was conducive to decreasing the peak temperature. In addition, when the temperature at night dropped, the heat stored during the day was released, thus increasing the night temperature. It could be seen from Figure 10 that the temperature fluctuation of the PCM room had decreased significantly. Therefore, the thermal comfort of the PCM room was greatly improved compared with the reference room. Moreover, the integration of PCMs into prefabricated temporary houses also contributed to the reduction in energy consumption. According to Table 8, the energy consumption of the reference room and PCM room was 2775 and 2656 kWh, respectively. The energy consumption of the PCM room was 119 kWh less than that of the reference room, and the energy saving rate was about 4.3%. In general, the PCM panel had the potential for energy conservation in the application of prefabricated temporary houses.

## 5. Conclusions

In this paper, composite PCMs with tunable phase transition temperature were prepared. DHPT was blended with DHPD to form eutectic hydrated salts. SiO_2_ and Na_2_SiO_3_·9H_2_O were used as the adsorption carrier and nucleating agent of eutectic hydrated salts, respectively. The properties of composite PCMs were tested and characterized, and their application performances were studied. The main conclusions were as follows:

1. Adding 15% deionized water in the preparation of eutectic hydrated salts was helpful in improving the thermal storage performance of PCMs. Na_2_SiO_3_·9H_2_O effectively reduced the supercooling degree of the composite PCM. When its content was 5%, the supercooling degree was 1.2 °C.
2. The maximum adsorption capacity of SiO_2_ on eutectic hydrated salt is about 70%. The SEM and FTIR test results confirmed that eutectic hydrated salts and SiO_2_ were combined, and the combination did not generate new substances. The TGA test results showed that the PCMs had good thermal stability, and the content of eutectic hydrated salts calculated according to the TGA results was basically consistent with the theoretical value.
3. With the increase in the content of DHPT, the melting enthalpy and phase transition temperature of composite PCMs decreased gradually. When the content of DHPT was 3%, the melting enthalpy of the composite PCM was 145.3 J/g, and its phase transition temperature (26.5 °C) was suitable for the field of building energy conservation. After 200 heating and cooling cycles, its melting enthalpy and phase transition temperature changed very little, which indicated that it had good thermal reliability.
4. The numerical simulation results showed that composite PCMs had good application performance in prefabricated temporary houses. By replacing the 10 mm insulation board with the 10 mm PCM panel, the thermal comfort hours of Grade I increased from 2187 h to 2991 h, and the energy consumption decreased from 2775 kWh to 2656 kWh.

## Figures and Tables

**Table 1:** The supercooling degree of different samples.

Sample	Mass Fraction (%)	Supercooling Degree (°C)
Deionized water	0	6.2
	5	7.0
	10	3.3
	15	2.8
	20	8.5
	25	6.0
DHPT	0	4.5
	3	5.4
	6	6.4
	9	6.9
	12	6.2
Na_2_SiO_3_·9H_2_O	2	3.7
	3	4.0
	4	3.4
	5	1.2
	6	3.6
	7	4.2

**Table 2:** Thermal characteristics of composite PCMs with different DHPT content.

Mass Fraction (wt%)	T_m_ (°C)	T_p_ (°C)	Melting Enthalpy (J/g)
0	29.6	35.0	150.3
3	26.5	33.0	145.3
6	25.1	32.3	136.6
9	23.4	31.5	133.3
12	22.9	30.7	122.4

**Table 3:** Thermal characteristics of composite PCMs before and after cycling.

Sample	Melting Process		
	T_m_ (°C)	T_p_ (°C)	Enthalpy (J/g)
Composite PCMs (before 200 cycles)	26.5	33.0	145.3
Composite PCMs (after 200 cycles)	27.1	33.7	139.3

**Table 4:** Comparison of thermal properties of composite PCMs.

Composite PCMs	Melting Point (°C)	Enthalpy (J/g)	Reference
CaCl_2_·6H_2_O-CO(NH_2_)_2_·/SiO_2_	21.0	114.3	[34]
Na_2_SO_4_·10H_2_O-Na_2_HPO_4_·12H_2_O/SiO_2_	25.2	142.9	[33]
Na_2_CO_3_·10H_2_O-Na_2_HPO_4_·12H_2_O/diatomite	24.1	102.6	[35]
Na_2_SO_4_·10H_2_O-Na_2_HPO_4_·12H_2_O/fly ash	25.3	106.9	[30]
Na_2_SO_4_·10H_2_O-Na_2_CO_3_·10H_2_O/expanded vermiculite	24.0	110.3	[19]
Na_2_HPO_4_·12H_2_O-K_2_HPO_4_·3H_2_O/SiO_2_	26.5	145.3	Present study

**Table 5:** Structural details of prefabricated temporary houses.

Envelope	Roof and Wall		Door	Floor	Window
	With PCM	Without PCM			
Outside layer	0.5 mm steel sheet	0.5 mm steel sheet	0.5 mm steel sheet	0.5 mm steel sheet	6 mm low-E glass
Layer 2	90 mm rock wool	100 mm rock wool	100 mm rock wool	100 mm rock wool	12 mm air layer
Layer 3	10 mm PCM panel	0.5 mm steel sheet	0.5 mm steel sheet	10 mm plywood	6 mm glass
Layer 4	0.5 mm steel sheet				

**Table 6:** Thermophysical properties of the building materials.

Material	Density (kg·m^−3^)	Thermal Conductivity (W·m^−1^·K^−1^)	Specific Heat Capacity (J·kg^−1^·K^−1^)
Steel sheet	7800	16.3	500
Plywood	600	0.17	2510
Rock wool	80	0.041	1220
PCM panel	1000	0.30	2100

**Table 7:** Evaluation grade of indoor thermal comfort.

Category	Indicator
Grade I	PDD ≤ 10% and −0.5 ≤ PMV ≤ +0.5
Grade II	10% < PPD ≤ 25% and (−1 ≤ PMV < −0.5 or +0.5 < PMV ≤ +1)
Grade III	PDD > 25% and (PMV < −1 or PMV > +1)

**Table 8:** Thermal performance of prefabricated temporary houses.

Room Type	Thermal Comfort Hours (h)			Energy Consumption (kWh)
	Grade I	Grade II	Grade III	
Reference room	2187	945	5498	2775
PCM room	2991	1136	4521	2656
